# Reply to “Radiation exposure and estimated cancer risk in neonates: a cautionary perspective”

**DOI:** 10.1007/s00330-024-11120-9

**Published:** 2024-10-28

**Authors:** Deliah Weiß, Martin Beeres, Ulrich Rochwalsky, Thomas J. Vogl, Rolf Schlößer

**Affiliations:** 1 Paediatrics and Adolescent Medicine/Department of Neonatology, Clinic of the Goethe University, Frankfurt, Germany; 2 Institute for Diagnostic and Interventional Radiology, Clinic of the Goethe University, Frankfurt, Germany; 3 Clinic for Neuroradiology, Marburg University Hospital, Marburg, Germany

First, we want to thank the authors for their view and valuable input to our manuscript [1].

The decreasing radiation exposure is driven by radiation-free imaging as well as upcoming techniques to gain information about, for example, correct catheter placement [2].

One recent study also underlines the risk of radiation exposure in children who received CT examinations in a traumatic background [3].

However, displacement of catheters is common in clinical practice, and X-ray remains a well-established method to control correct catheter placement [4].

We wanted to shed some light on the overall usage of conventional X-ray. Upcoming risk awareness is to some point absolutely legitimate, but it should not lead to an under-usage of a robust diagnostic tool such as chest X-ray imaging when necessary. The risk of a misplaced catheter with incorrect injection of medication that might be necessary to support the cardio-/vascular system outweighs the risk of X-ray, in our point of view.

If trained physicians in ultrasound are able to perform a correct ultrasound diagnosis with reliable confidence, this is the way to go; we absolutely agree. However, if these trained physicians are not available or other problems occur, conventional X-ray imaging might be the secure and non-user-dependent way to control inserted catheters as well as pulmonary or abdominal pathologies.

We agree that radiation-free imaging is a good choice and should be used more. If not available due to any circumstances, conventional X-ray remains a good opportunity to help cure our patients.
